# Supplementary material for: Community emergency management policy in China using a policy text tool
Source: PLoS One. 2025 Feb 25;20(2):e0319239. doi: 10.1371/journal.pone.0319239 (PMC11856356; doi:10.1371/journal.pone.0319239)
Supplement: Attachment — Submitted filename: Response to Reviewers.docx [file pone.0319239.s001.docx]

We sincerely thank the editor and all reviewers for their professional comments on our articles, which helped us improve our manuscript. The point-by-point response to the comments is given below.

**Comments from the editor：**

**General Comments:**

The editor agrees with the two reviewers that the paper does have its scientific merit in advancing the understanding of emergency management policy in China. Yet, the paper needs a thorough revision to increase its quality and paper organization before it can be accepted for publication at PLOS One.

**Specific comments:**

1.First of all, the paper needs to have a much better discussion of their methodology. There are multiple methodlogical components of the study, including text selection, content analysis, (social?) network analysis, policy analysis, etc. It is warranted that the authors create an overarching framework (or a flowchart at the higher level) that integrates these parts together (along with input data, and outputs/policy recommendations). The paper in its current form is very fragmented and needs a methodological framework as its backbone.

Answer: We agree. We think this is a good suggestion. By building an overall framework to integrate the methods used, the context of the article can be clearer. Therefore, we have created a general research framework, and put it in Part 3.1 of this paper, and added a frame diagram (see Fig. 1)(Line. 132-148).

2.Secondly, please make thorough check on the methodology description and make sure they are all adequately discussed. For instance, the content analysis and (social?) network analysis need more discussion and technical details. Also, arguably the method is no longer a social network problem but rather you used network and graph to represent different linkages of texts/words, if the Editor understood it correctly. Then, such a generalization needs to be formulated and discussed in the paper.

Answer: We agree. We have thoroughly checked the method description according to the editor's suggestion, and added more discussion and technical details to the research method in part 3.3 of the paper, and added an exposition of the use of network and graphic to express the relationship between keywords in social network analysis. (Line.176-179; 186-194; 312-317)

3.Third, please revise the policy recommendation and embed them in your Section 5. This is because many of your recommendations are backed up by your analysis findings, they should be discussed inline with your anlaysis results/visualizations. Evidences should then be cited to support each policy implication/recommendation item. In Section 6, you merely revisit these recommendations.

Answer: We agree. We have revised the policy recommendations according to the editor's suggestions and embedded them in Part 5, discussed them together with the research results, and added references to supporting evidence, and revisited these policy recommendations in Part 6. (Line.332-344; 366-368; 386-388; 400-408; 423-427; 434-438; 444-448; 470-474; 484-488; 502-505; 513-515; 519-552)

4.Last but not least, the paper lacks a discussion of contribution. Please highlight your innovation, whether it is within technical/methodological frame, or about empirical findings/policy suggestions, the readers should be informed on why this article is cutting-edge and has the value for publication at an impactful journal such as PLOS One.

Answer: We agree. We have emphasized our innovation and the value of the article in the research framework and policy suggestions. (Line.133-136; 554-557)

**Comments from the reviewers:**

**Reviewer 1**

**General Comments:**

The research of community emergency management in China is a field worthy of attention, and the selection of this field is worthy of full affirmation. Based on the analysis of policy texts, this paper puts forward reasonable suggestions for community emergency management in China, which is also worthy of affirmation. On the whole, the article is worth publishing. However, it is also necessary to pay attention to the following problems:

**Specific comments:**

1. First, there are too few policy text names listed in Table 1, and the policy text names directly related to the topic of this paper should be listed. The number of existing policy text names is small, and it is not clear which policy text is directly related to this paper.

Answer: We agree. We sincerely thank you for your valuable suggestions. We have added policy texts directly related to the theme of this article in Table 1, which makes the display of policy texts clearer. Such as “Opinions on Further Playing the Role of Emergency Broadcasting in Emergency Management”; “Opinions on Improving and Perfecting the Comprehensive Service Functions at the Village Level” , etc.(Line.166)

2.Second, there are many errors in the language of this paper. It is recommended that professional service organizations make comprehensive changes to the text language.

Answer: We agree. Thank you for your advice. We found a professional organization (AJE) to polish our article, and we tried our best to revise it. These changes will not affect the content and framework of the paper, so we didn't list the changes here. In addition, according to the suggestion of AJE, we revised the title of the article to “Community Emergency Management Policy in China Using a Policy Text Tool”. We uploaded the polished version to the supporting information, and we hope that the revised manuscript will be accepted by you. (Line.1-2)

**Reviewer 2**

1.Abstract: "community emergency management policy (CEMP)" should be changed to "Community Emergency Management Policy (CEMP)" for consistency.

Answer: We agree. We have made corresponding changes in the abstract to maintain consistency. (Line. 11)

2.Line 13: There is an inconsistency between "two dimensional analysis framework of 'policy tool-policy goal'" and line 128: "two-dimensional analysis framework of 'policy tools-policy objectives'." These terms should be unified.

Answer: We are very sorry for our careless mistakes. Thank you for reminding us. We have unified the terms in these two places. (Line.15 and Line126)

3.Line 143: The word "plans" is duplicated.

Answer: We are very sorry for our careless mistakes. We have deleted the redundant words.(Line.154)

4.Line 482: In the 6.2 Policy Recommendations section, I suggest adding steps for emergency management evaluation. Based on the evaluation results, identify the effectiveness of policy tools and then give recommendations according to their effectiveness. The emergency scenario should be considered when evaluating the effectiveness of policy tools.

Answer: Thank you for your suggestion. We have added relevant explanations to the assessment in the emergency management assessment step, and suggested that emergency scenarios should be considered. (lines 533-538) .

5.On Line 140, would using "community emergency" as a keyword provide more comprehensive data results?

Answer: We sincerely thank you for your careful reading. We tried to use "community emergency" as the key word to search. In addition to the policy texts already covered by other key words, we supplemented the policy text "Opinions on Further Improving the Emergency Management Capability at the Grass-Roots Level" (2024) and listed it in Table 1. (Line.158 and Line.166)

6.On Line 141, why did you choose those three keywords? Several other keywords, such as "emergency response," "emergency rescue," and "emergency drills," are mentioned frequently in the paper. Why were they not selected?

Answer: We sincerely thank you for your careful reading. The keywords we selected cover the policy texts about community emergency management, but we still try to search carefully again with keywords such as "emergency response", "community emergency rescue" and "community emergency drill", and supplement the policy texts by adding five policy texts such as "People's Republic of China (PRC) Emergency Response Law" (2024). As shown in Table 1. (158-159; 166)

In addition, we extended the policy text retrieval time from 2004-2023 to 2004-2024. At the same time, after adding relevant search keywords, the number of policy texts increased from 81 to 87. "5.Quantitative analysis of CEMP Texts" has updated the corresponding charts and data in the text.(See Fig3; Fig5; Fig6; Fig7 ) (Line.272; 324-327; 348; 354; 363-364; 379; 395-397; 414-416; 420-421; 431-432; 441; 455-456; 461; 466-467; 476-477; 490-493; 500;507-508)

7. On Line 216, are the tools listed in Table 2, "Division of CEMP Tools," sourced from the Magic Weapon Legal Database of Peking University?

Answer: Clarification. Thank you for your question. The tools classification listed in Table 2 "Division of CEMP Tools" adopts Rothwell and other policy tools classification standards. See Reference [34] for details.(Line.211-213; 223-225)
